# Glucometrics utilisation in an urban teaching hospital in ireland: current practice and future aims

**DOI:** 10.1007/s11845-024-03768-5

**Published:** 2024-08-05

**Authors:** Joseph McGauran, Arianna Dart, Phyllis Reilly, Matthew Widdowson, Gerard Boran

**Affiliations:** 1 Trinity College, Dublin, Ireland; 2 Tallaght University Hospital, Dublin, Ireland

**Keywords:** Diabetes mellitus, Dysglycaemia, Glucometer, Point-of-care-testing

## Abstract

**Background:**

Dysglycaemia in hospitalised patients is associated with poorer clinical outcomes, including cardiovascular events, longer hospital stays, and increased risk of mortality. Therefore, glucose monitoring is necessary to achieve best outcomes.

**Aims:**

This audit assesses use of point-of-care (POC) blood glucose (BG) testing in Tallaght University Hospital (TUH) over an 8-day period. It evaluates compliance with international and TUH glucose monitoring protocols and determines frequency of diabetes team consultations for inpatient adults.

**Methods:**

Data from an 8-day period (12/03/2023–19/03/2023) were extracted from the TUH COBAS-IT system and analysed. Invalid tests were excluded. Hyperglycaemia was defined as ≥ 10 mmol/L and hypoglycaemia as ≤ 3.9 mmol/L. Persistent hyperglycaemia was defined as two BG results of ≥ 10 mmol/L. A chart review was conducted on adult patients with persistent hyperglycaemia to assess for HbA1C results, diabetes diagnosis, and diabetes consult.

**Results:**

3,530 valid tests were included and analysed. 674 individual patients had tests done. 1,165 tests (33.00%) were hyperglycaemic and 75 (2.12%) were hypoglycaemic. 68.25% of adults with persistent hyperglycaemia had an HbA1C test performed or documented within three months. 42.71% of inpatient adults with persistent hyperglycaemia and a known diabetes diagnosis received a consult from the diabetes team.

**Conclusion:**

Increased adherence to hospital protocols for testing HbA1C in adults with persistent hyperglycaemia could improve treatment and clinical outcomes. Increased diabetes team consultation could facilitate appropriate treatment and improve patient outcomes in persistently hyperglycaemic adult patient populations.

## Introduction

### Dysglycaemia

Blood glucose (BG) level homeostasis is necessary for normal physiological function and both hyperglycaemia and hypoglycaemia have notable adverse effects. Hyperglycaemia is defined by the American Diabetes Association (ADA) and American Association of Clinical Endocrinologists as a BG > 7.8 mmol/L [1]. Persistent hyperglycaemia can have many adverse effects, including micro and macrovascular complications [2]. Microvascular complications include the ‘microvascular triad’ of retinopathy, nephropathy, and neuropathy. Macrovascular complications include stroke, coronary artery disease and peripheral arterial disease [2]. These vascular complications of hyperglycaemia are a major source of morbidity and mortality in Type I Diabetes Mellitus (T1DM) and Type II Diabetes Mellitus (T2DM) [3]. Hyperglycaemia can also result in hyperglycaemic emergencies such as Diabetic Ketoacidosis (DKA) and Hyperosmolar Hyperglycaemic Syndrome (HHS), with DKA occurring mainly in T1DM and HHS in T2DM [4]. In addition to DKA and HHS, inpatient hyperglycaemia is also associated with poor clinical outcomes in general [5]. Hypoglycaemia is defined by the ADA as a BG level lower than 3.9 mmol/L [6]. It is further classified into Level 1 (hypoglycaemia alert), Level 2 (clinically significant hypoglycaemia), and Level 3. Level 1 hypoglycaemia is a BG level between 3.0–3.9 mmol/L. Level 2 is a BG level below 3.0 mmol/L. Level 3 does not have a specific glucose level but is measured clinically; it is characterised by cognitive or physical impairment requiring external assistance for recovery. Both Level 2 and Level 3 require prompt medical attention [7]. Hypoglycaemia can lead to serious sequelae, including seizure, loss of consciousness, and coma [8]. Causes of hypoglycaemia include excessive insulin dosing or inappropriate timing of insulin administration with food intake; errors in medication orders or administration [9]; administration of systemic steroids [10]; polypharmacy; and delayed or missed meals [11]; and organ failure, such as liver or kidney failure [12].

### Dysglycaemia in hospitalised patients

Treating both hyperglycaemic and hypoglycaemic patients is vital for improving outcomes in hospitalised patient populations. A retrospective study of over 250,000 ICU-admitted veterans in the US found that hyperglycaemia with or without a diagnosis of diabetes was “independently associated with increased mortality after adjustment for severity of illness” [13]. The same study also noted an increased risk of death proportional to increasing BG levels [13]. The same is also seen in non-ICU hospital settings [5]. Hypoglycaemia is associated with both increased length of stay in hospital and higher mortality during hospitalisation [14]. In fact, hypoglycaemia in the ICU population was found to be an independent risk factor for mortality [12]. Hypoglycaemia is a risk factor for cardiovascular events [15], including cardiac ischemia [16]. It is also a risk factor for falls during hospitalisation [17], particularly in older populations [18].

### The importance of monitoring blood glucose

In order to prevent dysglycaemic events and treat them appropriately when they do occur, it is necessary to identify at-risk patients promptly. This can be difficult because both hyperglycaemia and hypoglycaemia are often asymptomatic [11, 19]. Therefore, it is imperative that BG levels of hospitalised patients are frequently monitored. This is especially critical in diabetic patient populations, as they are most at risk of hyperglycaemia and iatrogenic hypoglycaemia. Point-of-care (POC) BG testing is an effective way to monitor patient BG levels [20], and allows healthcare providers to identify cases of dysglycaemia, thus enabling timely intervention and prevention of the adverse outcomes associated with both dysglycaemic states. However, it must be noted that these POC devices can only be used for monitoring of blood sugar and not to diagnose diabetes, as per their licence. There are various protocols established at international, national, and local levels to ensure monitoring is done with appropriate timing and frequency. Although hyperglycaemia is defined as BG > 7.8 mmol/L [1], the consensus statement by the ADA and Clinical Practical Guidelines of the Endocrine Society recommend BG targets between 7.8 mmol/L and 10 mmol/L for diabetic patients [21, 22]. Although strict control of BG to < 7.8 mmol/L may be at times beneficial [23, 24], various other trials [25–27] have found no benefit in patient outcomes and have actually found increased risks associated with intensive glycaemic control such as the increased incidence of hypoglycaemic events. As such, targets for diabetic patients are set to 7.8—10 mmol/L [21].

### Value of diabetes team consultations

In addition to identifying dysglycaemic patients quickly, having specialised care teams consult them while in hospital can improve patient care, through improving glycaemic control [28], and reducing length of stay [29–31]. TUH has an inpatient diabetes team for adult patients, which aims to provide consults to both known diabetic patients and newly diagnosed patients. It is not known, however, what percentage of patients this team consults while they are in hospital or how quickly after at-risk patients are identified this team visits them.

### Aims of this analysis

As outlined above, dysglycaemia can result in poor patient outcomes. As such, it is important that BG levels are regularly monitored. In this audit, data were analysed from POC BG measurements in TUH over an 8-day period (12/03/2023 – 19/03/2023) to assess overall glucometer usage in TUH. As explained above, the ADA recommends BG targets between 7.8 mmol/L to 10 mmol/L for diabetic patients. Tallaght University Hospital (TUH) has slightly different protocols, with glycaemic targets for diabetic patients of 6—10 mmol/L, per the ‘Adult Medicine Guide.’ The ADA recommends a HbA1C test for diabetic and hyperglycaemic patients who have not had a HbA1C test done within three months [21]. Common practice at TUH is to perform a HbA1C on patients with persistent hyperglycaemia, defined as a BG of ≥ 10.0 mmol/L on two separate occasions if they have not had a HbA1C recorded in the last three months. Adherence to these glucose monitoring protocols in TUH, including monitoring for persistent hyperglycaemia and checking for HbA1C in persistently hyperglycaemic patients, were investigated. Inpatient consults of persistently hyperglycaemic adult patients by the TUH inpatient diabetes consult team were also assessed.

## Methodology

The glucometer currently used in TUH for POC BG testing is the Accu-Check Inform II from Roche Diagnostics, Basel, Switzerland. There are 120 of these devices [32] placed on all hospital wards and used by trained care providers. The data collected from these devices are connected to the COBAS-IT system [33], which generates a central database managed by the Laboratory Medicine Department.

Data were extracted from the COBAS-IT glucometrics system in TUH from an 8-day period (12/03/2023—19/03/2023). The data were anonymised by patient medical record number (MRN) and exported to Microsoft Excel (Version 16.71), where it was analysed. Only valid glucose readings were included in this analysis. Glucose readings were deemed invalid and therefore were excluded for the following reasons: no MRN entered on the glucometer before use, incorrect MRN entered, or unrecognised MRN because the patient was not clerically admitted to the area where the sample was processed.

Analysis was done to assess how many patients with a BG test of ≥ 10 mmol/L had their BG levels rechecked to monitor for persistent hyperglycaemia, defined as two BG readings of ≥ 10 mmol/L. The number of patients with persistent hyperglycaemia was calculated.

A chart review was conducted on adult patients with persistent hyperglycaemia. Paediatric patients were excluded from this portion of the analysis due to lack of adequate access to paediatric patient charts. Adult patients were isolated by excluding patients with BG readings taken by glucometers on paediatric wards. HbA1C results and whether or not the patient had a previous diagnosis of diabetes were extracted from the patients’ medical records. For adult inpatients, it was also determined whether or not the patient was seen by the TUH in-hospital diabetes team during their hospital stay. Paediatrics were excluded from this analysis because the inpatient diabetes team only consults adult patients. Outpatients were excluded based on glucometer location; all patients who had BG readings only from glucometers in outpatient settings or only from the dialysis day unit were removed from this portion of the analysis.

## Results

### Number of tests done

**Fig. 1:**
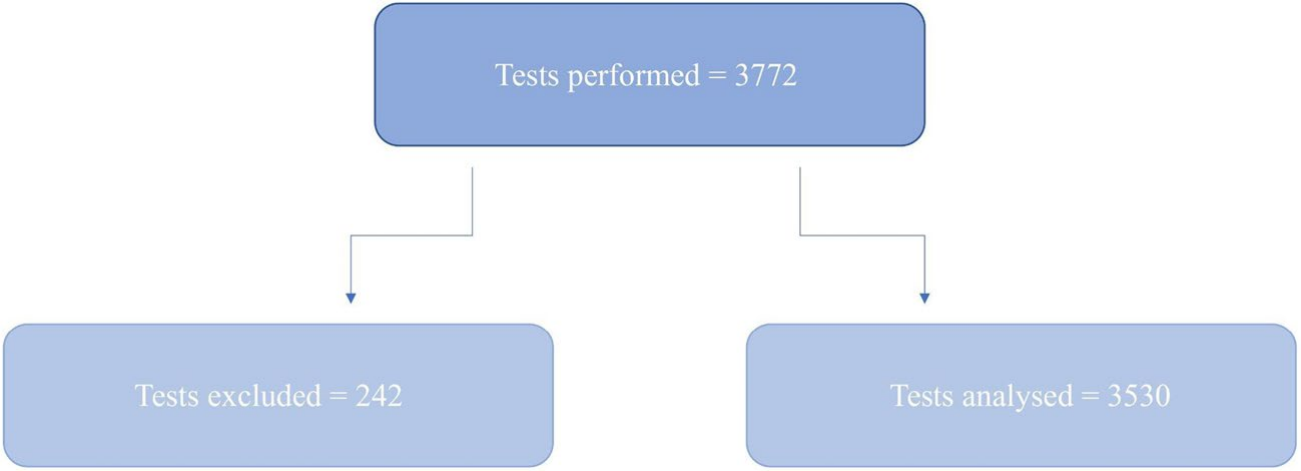
Number of tests included and excluded from analysis

A total of 3,772 POC BG tests were performed. Of these, 242 were invalid and excluded. As such, the error rate for this 8-day period was 6.42%. Ultimately, 3,530 tests were included Fig. 1.

674 individual patients had their BGs measured in the audit’s 8-day period. Of the 674 individual patients, the number of POC BG tests performed per patient ranged from 1 to 87.

### Dysglycaemic test results

1,165 readings were ≥ 10 mmol/L (33.00% of tests performed). The number of individual patients with at least one POC BG test ≥ 10 mmol/L was 216 (32.05% of patients). 75 hypoglycaemic events ≤ 3.9 mmol/L occurred in this audit (2.12% of tests performed).

### Monitoring for persistent hyperglycaemia

Of the 674 individual patients identified, 216 of them had a BG test ≥ 10 mmol/L during their time in hospital. Of these, 177 (81.94%) had their BG rechecked. 39 (18.06%) did not have a BG recheck. Of the 177 with their BG rechecked, 130 (60.19% of 216) had persistent hyperglycaemia.

### HbA1C levels in adult patients with persistent hyperglycaemia

**Fig. 2:**
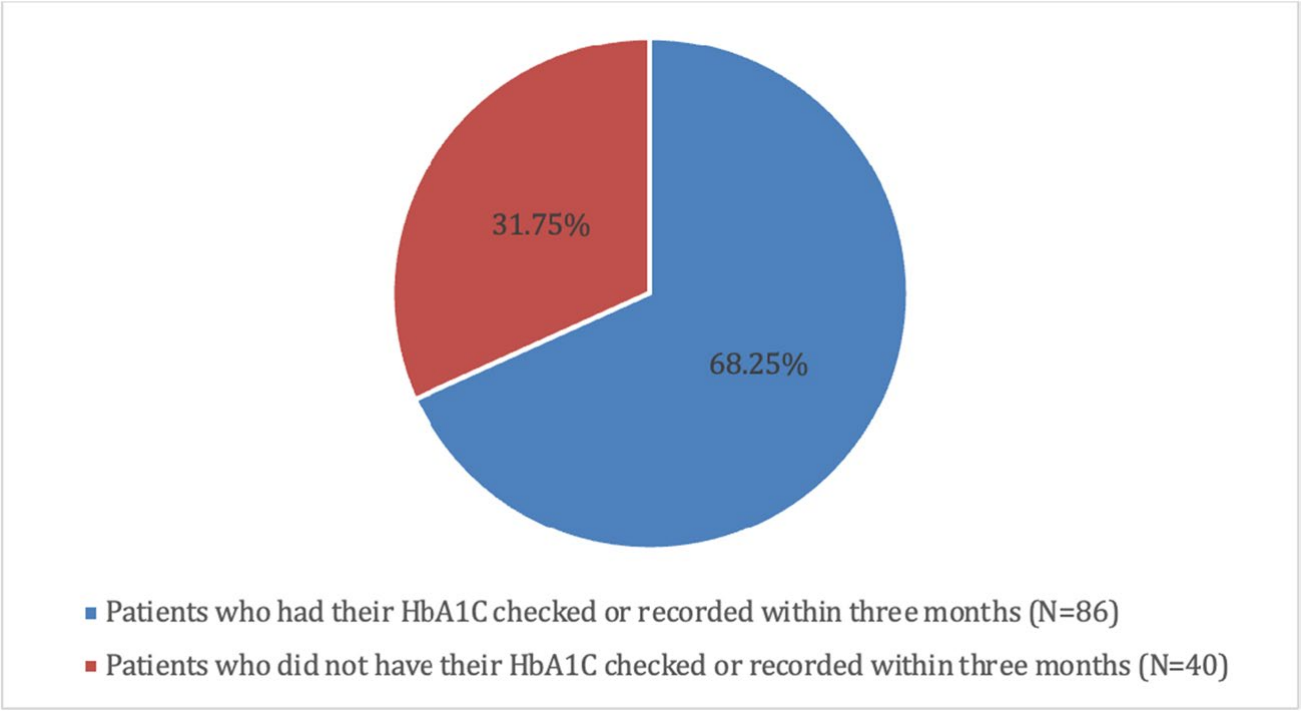
Percentage of adult patients with persistent hyperglycaemia who had an HbA1c checked in hospital or recorded within three months of hospitalisation

Of the 130 patients identified with persistent hyperglycaemia, 126 were adults. Of these 126 adult patients, 86 (68.25%) had their HbA1C levels checked either in hospital or had a record of a test done in TUH within the previous three months Fig. 2.

### Diabetes diagnosis and consultation in adult patients with persistent hyperglycaemia

**Fig. 3:**
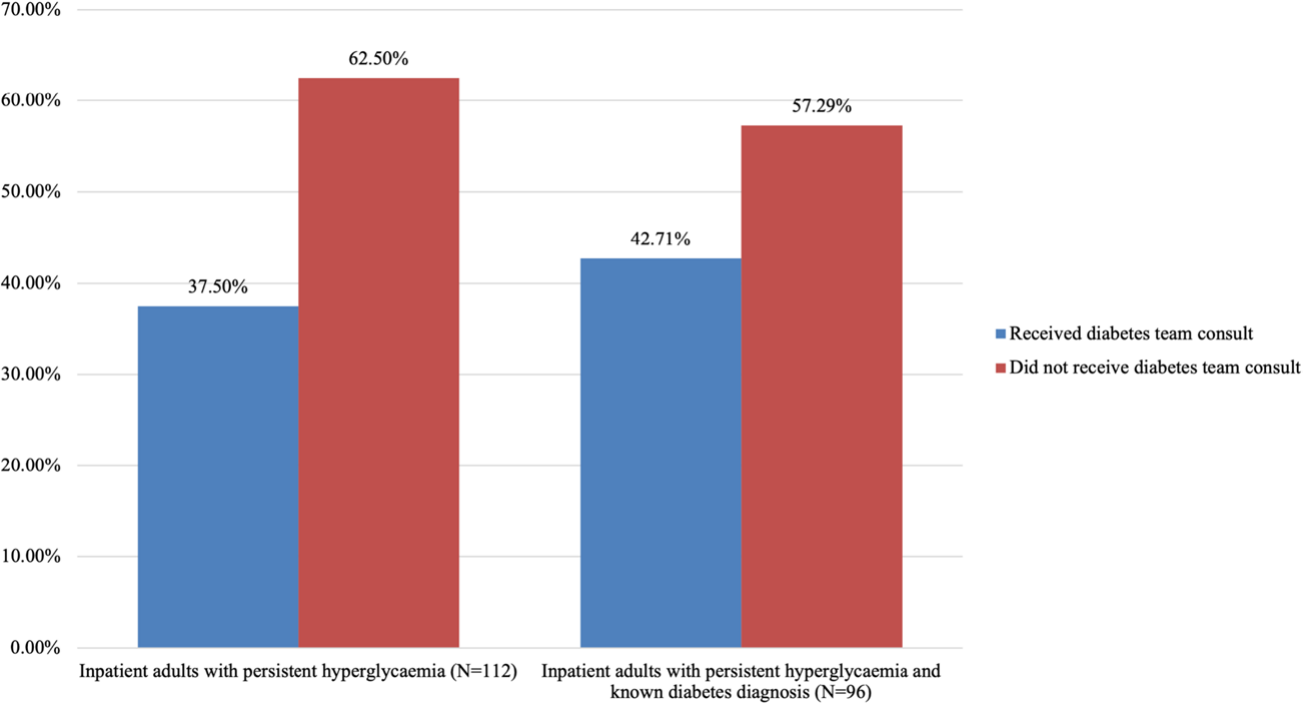
Percentage of patients with persistent hyperglycaemia who received a diabetes team consult

Of the 126 adult patients with persistent hyperglycaemia, 112 were inpatient. Of these, 96 (84.82%) had known diabetes diagnoses. 83 (86.46% of 96) had Type II DM, 11 (11.46% of 96) had Type I DM, 1 (1.04% of 96) had Type IIIc DM, and 1 (1.04% of 96) was stated to have “diabetes” but the type was not specified. Of the 112 adult inpatients with persistent hyperglycaemia, 42 (37.50%) were seen by the diabetes consultation team in TUH during their hospital stay and 70 (62.50%) were not. Of the 96 known diabetic patients with persistent hyperglycaemia, 41 (42.71%) were seen by the diabetes consultation team during their hospitalisation and 55 (57.29%) were not Fig. 3.

## Discussion

### Adherence to guidelines for glucose monitoring

Patients with a BG reading of ≥ 10 mmol/L should receive a repeat BG check to assess for persistent hyperglycaemia. In this audit, 18.06% of those with a BG reading of ≥ 10 mmol/L did not receive a repeat test. The ADA recommends prompt insulin therapy be initiated in patients with persistent hyperglycaemia [21]. In order to identify these patients and ensure proper intervention, repeat BG tests must be done on all patients with a single reading of ≥ 10 mmol/L. Furthermore, over 30% of adult inpatients with persistent hyperglycaemia did not have a HbA1C performed in hospital and did not have one within 3 months on record, although protocol recommends it. These findings imply that the monitoring of patients with hyperglycaemia in TUH may be improved. This could be achieved with updated staff training, improving quality checks, and implementation of an electronic alert system for dysglycaemic events, described below.

### Inpatient diabetes consults

This analysis showed that 62.50% of adult inpatients with persistent hyperglycaemia were not seen by the diabetes consult team at the time of the audit. Of the adult inpatients with persistent hyperglycaemia and a known diabetes diagnosis, 57.29% did not receive a consult from the diabetes team during their stay. A literature review on the topic found that in-patient diabetes management service is associated with hospital cost savings through decreased length of stay, decreased re-admissions and decreased hypoglycaemic events [31]. It is as a result advisable that TUH increase the utilisation of their inpatient diabetes consult team for these higher-risk patient populations. Additionally, the timeliness of the consultation may also be important. At the time of the chart review, many patients were still admitted to TUH and had not yet had a consult. Therefore, it is possible that the team did conduct a consultation with these patients after the chart review had been completed and before the patient was discharged. However, the analysis was conducted at least 7 days and at most 15 days after the patient became persistently hyperglycaemic. Thus, even if the team did ultimately consult a patient after the chart review, there would have been a significant delay between the time the patient was identified as persistently hyperglycaemic and the time they received a consultation. Such a delay would not contribute to immediate improvement in glycaemic control, nor to a shorter hospital stay for that patient. It is recommended that in addition to utilising the inpatient diabetes consult team more frequently, guidelines be established about how quickly this consult team should meet with patients during their hospital stay.

### Glucometrics system utilisation

The current TUH glucometrics system has a central database updated in real-time with all POC BG levels taken in the hospital. Currently, these data are utilised for research purposes only, and it is not used clinically by physicians for the monitoring and management of dysglycaemic patients. We propose that a computerised alert system for dysglycaemic events be introduced in TUH using the COBAS-IT system already in place in the hospital. Real-time dysglycaemia alerts have shown to result in better glucose control [34]. Another study also found that a computerised hospital hypoglycaemia alert system decreased severe hypoglycaemia by 68% in high-risk patient populations [35]. Given these positive outcomes, it is advised that an alert system is put in place in TUH, and that an audit is performed to check its effectiveness and clinical utility. A number of hospitals in Ireland also utilise Roche Accu-Check Inform II Glucometers and the COBAS-IT glucometrics system. Similarly to TUH, these glucometrics systems are not used clinically to identify dysglycaemic patients. However, because the glucometrics system is in place in these hospitals, if an alert system in TUH were successful, it could feasibly serve as a model for implementation of similar alert systems in hospitals across Ireland.

### Limitations of this audit

First, this audit only looked at data from an 8-day period. More robust analysis would include a longer timeframe. Second, it was not possible to ascertain whether or not specific BG levels were pre or post-prandial. As such, cut-offs for hyperglycaemia were not as specific as possible. Finally, when assessing whether or not inpatient adults received consultation from the diabetes team in TUH, patients who had not yet been discharged at the time of the chart review were not excluded. As such, it is possible that a diabetes consult may have occurred after the chart review was conducted and this would not have been captured.

## Conclusion

BG monitoring is essential to identifying patients with dysglycaemia and facilitating timely intervention. There are various guidelines outlining best clinical practice for BG monitoring. Those implemented in TUH are available in the ‘Adult Medicine Guide.’ This analysis shows that identifying persistent hyperglycaemia and checking HbA1C in persistently hyperglycaemic patients could be improved in TUH. Good compliance with these guidelines could lead to better glycaemic control and patient outcomes. We propose an electronic alert system be put in place in TUH to decrease the workload burden of clinical staff and improve prompt detection of dysglycaemic patients. Furthermore, this analysis showed that many diabetic and persistently hyperglycaemic inpatients did not receive consultation from the inpatient diabetic team. Therefore, we recommend that TUH work to increase the efficacy and utilisation of the inpatient diabetes consult team to best care for dysglycaemic patients.

## Declarations

### Audit registration

To comply with policy at TUH, the audit was registered as an audit on the TUH/SJH Research Ethics Committee Infonetica System, registration number 4503.
